# The effect of antimicrobial activity of *Teucrium Polium* on Oral *Streptococcus Mutans*: a randomized cross-over clinical trial study

**DOI:** 10.1186/s12903-020-01116-4

**Published:** 2020-05-01

**Authors:** Somayeh Khoramian Tusi, Ahmad Jafari, Seyed Mahmoud Amin Marashi, Salomeh Faramarzi Niknam, Malihe Farid, Mehdi Ansari

**Affiliations:** 1 Department of Pediatric Dentistry, School of Dentistry, Alborz University of Medical Sciences, Alborz, Iran; 2 Research Center for Caries Prevention, Dental Research Institute, Department of Community Oral Health, School of Dentistry, Tehran University of Medical Sciences, Tehran, Iran; 3 Department of Pediatric Dentistry, School of Dentistry, Al Hussain University, Karbala, Iraq; 4 Department of Microbiology, Qazvin University of Medical Sciences, Qazvin, Iran; 5 Department of Community Medicine, School of Medicine, Alborz University of Medical Sciences, Alborz, Iran; 6 Department of Pharmaceutics, Faculty of Pharmacy, Kerman University of Medical Sciences, Kerman, Iran

**Keywords:** Mouthwash, Medicinal plants, Salvia, Herbal extract, Dental caries, Oral hygiene

## Abstract

**Background:**

The purpose of this study is to determine the effect of a mouthwash containing *Teucriumpolium* herb on *Streptococcus mutans* in mouth.

**Methods:**

This study was a randomized, crossover, double-blind clinical trial, where we selected 22 volunteers (dental students) randomly and we divided them into two groups. The study had two phases. In each phase, one group acted as the intervention group, while the other one was the control group. Both the intervention and control groups were given the mouthwash with and without *Teucriumpolium*, respectively. *S. mutans* of saliva were measured before and after each phase to compare the effects of the mouthwashes. A three-week washout period was considered between the two phases. An independent two-sample t-test was utilized to compare the mean of *S. mutans* colonies. Additionally, we used a standard AB/BA crossover model to find the results of the treatment and the impact of carryover on the residual’s biological effects. The significance level was considered 0.05 in this experiment.

**Results:**

There is no significant difference observed between the two groups in the number of *S. mutans* before using the mouthwashes. When the mouthwash containing *Teucriumpolium* was used, there was a significant decrease in the number of *S. mutans* colonies in both phases’ extract (*P* = 0.002). *Conclusion*: The results of this study indicate the mouthwash containing aqueous extract of *Teucrium polium* can majorly reduce the colonization of *S. mutans* in human saliva.

**Trial registration:**

Ethical issues approved by the Ethics Committee of the Rafsanjan University of Medical Sciences with the approval number of 937/9/31, IRCT code Number of IRCT2013121815842N1 and it was approved on 06/16/2014. The study was conducted in the period of September to November 2014.

## Background

A healthy mouth is the gate to a healthy body. The effects of oral health on many systemic diseases and cancers are extensively studied and there are some proven theories [1–3]. Amongst a wide variety of microorganisms, *Streptococcus* pathogens, such as *Streptococcus mutans* (*S. mutans*), are worth mentioning. They can result dental caries, while they can also deteriorate the general health and oral hygiene of a human being. Ultimately, these bacteria can impose huge medical expenses or bring about teeth loss. Therefore, finding the substances that have the capability to remove or to minimize these microbial species is of high importance in respect to this subject. In recent years, herbal medicines are receiving a great attention (based on the knowledge of traditional medicine) to prevent and to treat various illnesses. Dental science keeps up with the other medical specialties to utilize herbal and traditional medicine in the realm of oral health too. Some studies investigate the effect of medicinal plants as antibacterial agents in dentistry [4, 5], while herbal extracts are widely used in oral hygiene products as well. In some studies on mouthwashes, chlorhexidine is used as a positive control to compare the efficacy of other products, and is believed to be superior to other chemical ingredients such as listerine and betadine [6]. However, the incidence of such side effects as undesirable tooth discoloration, unpleasant taste, dryness, and burning sensation in the mouth might discourage patients to use mouthwashes containing this ingredient [7]. To overcome such side effects, nontoxic herbal mouthwashes using various herbs and plant extracts have been introduced.

Polei-gamander, with the scientific name of *Teucriumpolium* (*T. polium*), is a kind of peppermint that is a component of aromatic plants. It contains some organic materials with antibacterial effects [8]. *T. polium* or Polei-gamander is one of the medicinal herbs in traditional medicine, which has been used cancer treatments [8], gastrointestinal disorders [9], kidney and kidney stones [10], wound healing [11], and diabetes [12].

In dentistry, *T. polium* is consumed to produce probiotic compounds containing herbal extracts [13]. Antimicrobial mouthwashes work in a variety of ways, including the connection to the bacterial cell wall and the destruction of the wall. However, there is an immediate need to conduct extensive researches in this realm. Such studies will assist us in understanding the mechanism of such antibacterial activities as well as a better perspective of *T. polium‘s* effect on *S. mutans*. As a result, the purpose of the current research is to investigate the effect of a mouthwash containing *T. polium* herb on the number of salivary *Streptococcus mutans*.

## Methods

**Fig. 1:**
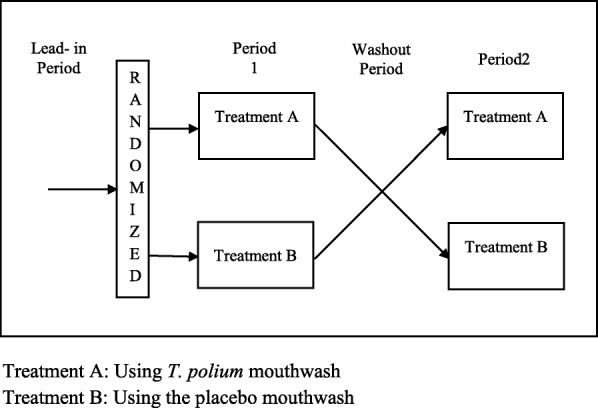
AB/BA randomized, crossover study design. Treatment A: Using *T. polium* mouthwash. Treatment B: Using the placebo mouthwash

An AB/BA randomized, crossover, double-blind clinical trial study was designed (Fig. 1).The Ethics Committee of the Rafsanjan University of Medical Sciences approved the ethical issues. The approval number is 937/9/31 and with the reference code of IRCT2013121815842N1 from the Iranian Registry of Clinical Trials (IRCT).

### Inclusion criteria

Inclusion included four criteria: Not using antibiotics in the last month, Not having active caries, Plaque index equal to or less than 20% (demographics aging from eighteen to twenty-five years old) and signing a consent form before participating in this study.

### Samples

Samples were selected from dental students of Rafsanjan School of Dentistry. Sample size was calculated based on a previous study [14]. The following formula was used to estimate the sample size:

$$ {n}_1={n}_2=\frac{\sigma_d^2{\left({Z}_{1\frac{a}{2}}+{Z}_{1-\beta}\right)}^2}{2{\varDelta}^2}\left(\alpha =0.05\to {Z}_{1\frac{a}{2}}=1.96;\kern0.5em \beta =0.10\to {Z}_{1-\beta }=1.29;{\sigma}_d=5.61;\kern0.5em \varDelta =4\right) $$

Therefore: *n*_1_ = *n*_2_ ≈ 11. As a result, each group needed eleven samples.

Twenty-two dental students, who volunteered to participate, were randomly chosen and were put into two categories: Intervention group and control group. Each group included 11 individuals of both genders.

Number 1 (representing the intervention group) and number 2 (representing the control group) were written on twenty-two pieces of paper (11 each), and were placed in a bowl. We asked the participants to each take a number so they can be assigned to the group the number represented. Their groups were assigned based on the random selection of the papers. Each student was given a two-digit code (01 to 22). The codes were used during the trial, instead of their names.

The study had two phases. In each phase, one group was chosen as intervention, while the other group was the control group. Intervention and control groups were given the mouthwashes with and without *T. polium*, respectively. The codes of the students were written on mouthwash bottles and were given to the students, so that neither the students nor the laboratory personnel were aware of the type of mouthwash (double-blinded).

The volunteers were asked not to change their usual health care practices, but keep 15 mL of the mouthwash in their mouth for 30 s twice a day for two weeks. They, however, were requested to neither have food or beverages nor wash their mouthsfor almost 30 min [15]. After two weeks, they entered a 3-week washout phase (not using mouthwash). This exercise wasfor saliva’s *S. mutans* value to return to initial levels [16]. In this phase, opposite to the first phase, placebo and *T. polium* mouthwashes were given to the first and second groups. Then, they were asked not to change their usual health care habits again, but keep 15 mL of the mouthwash in their mouth for 30 s twice a day for two weeks.

### Mouthwash preparation

The extract of *T. polium* was prepared at the Faculty of Pharmacy, Kerman University of Medical Sciences.*T. polium* was collected from the mountainous area around Kerman, the capital city of Kerman province in the South East of Iran, in June. Plant flowers were cleaned, then washed with cold water and deionized water (Zolal Teb Shimi, Tehran, Iran), and were dried in a place away from direct sunlight. The dried samples were grounded with an electric mill (Moulinex 1043, Paris, France), and passed through a sieve with a mesh size of 32. Next, 250 g of the powder was soaked in 2 l of water for 48 h and wasfiltered with a vacuum pump (Eyela A-35, Tokyo, Japan), a Buchner funnel (Isolab, Hannover, Germany), and a filter paper (Munktell, Bavaria, Germany). Obtained extracts were concentrated by vacuum distillation method at 52° Celsius, using a rotary machine (Lab Tech EV 311-V, Rome, Italy). Eventually, concentrated extracts were dried in an oven (Memmert UF-55, Frankfurt, Germany) at 42°Celsiusfor 3 days. Extraction yield was 10%. Dried extract was kept in a freezer at − 22° Celsius for further experimentation.

All components in the mouthwash, except for *T. polium* extract, were utilized to prepare the placebo. Distilled water (Zolal Teb Shimi, Tehran, Iran) was used instead of *T. polium* extracts to make the placebo.

Prepared mouthwashes were packed in matte plastic containers, and thenwere tagged with specific codes that were only known to the researchers, and were given to the volunteers. Each compound was prepared at the earliest time to its consumption to ensure the highest drug stability and the highest amount of active ingredients. After extract preparation, 0.2% concentration of mouthwash was chosen for further experiment due to its adequate taste. The mouthwash ingredients were as below: 1 Liter deionized water, 2 g of *T. polium* extracts, 1 g of artificial sweetener powder of 0.1% aspartame (Merck, Darmstadt, Germany), and 5 g powder of 0.5% coffee flavor (Nestle, Netherland, Holland).

### Saliva sampling

To sample the volunteers’ saliva, they were asked not take food or beverage for almost one hour prior to sampling. Sampling was done every day at 10 AM while 2 ml of their unprovoked saliva was collected in a sterile container (spitting method) [17]. Unprovoked saliva sample of all individuals was taken at the beginning of the study, before using the mouthwash. The second test was conducted at the last day of the first phase of experiment using a spitting method. Participants were asked to take a minute’s saliva in their mouth, and then pour in a sterile falcon tub.The third and fourth tests were done before and after the second phase. During the experiment, the volunteers were asked not to change their daily health care practices and brush their teeth using Crest toothpaste and Oral B toothbrushes.

### Colony count

To determine the number of *Streptococcus mutans* colonies, samples were sent to the Laboratory of Microbiology, University of Rafsanjan, Iran. One day before saliva sampling, the culture medium was prepared. The TYCSB (Tryptone-Yeast-Cysteine-Sucrose-Bacitracin) medium of *S. mutans* was prepared in accordance with the standard protocol [18]. Standard 0.9% NaCl (Zolal Teb Shimi, Tehran, Iran) was used to make a 1:1000 dilution of the samples, and then they were transferred to the plates containing 20 ml agar TYCSB using a 0.01 ml standard loop (Lab Tron, Tehran, Iran) [19]. The plates were placed in a CO_2_ incubator (Gallenkamp, Munich, Germany) at 37° Celsius for 48 h.

Accordingly, Gram’s method was used to distinguish between gram negative and positive bacteria. Gram-positive colonies (cocci) were selected to perform the catalase test. Next, catalase-negative cocci were incubated under biochemical tests for 48 h at 37°Celsius. Based on previous recommendations [20, 21], colonies of positive mannitol, positive vogues-proskauer (VP), negative arginine, positive dextran, negative urease, and positive bileesculin were the target colonies. Eventually, *Streptococcus mutans*’ colonies were calculated and were multiplied by dilution ratio to determine the number of colonies per one milliliter of each participant’s saliva (CFU/mL).

### Statistical analysis

Data was analyzed by SPSS software (Version 18.0). Quantitative data was reported via the Mean value and Standard Deviation (numbers and percentages). Paired t-test was used to determine the effect of each mouthwash on the *Streptococcus mutans*’s colonies. Moreover, an independent two-sample t-test was used to compare the Mean of *Streptococcus mutans*’s colonies after mouthwash consumption in each phase of the experiment. Besides, crossover analysis was used to compare the mean of *Streptococcus mutans*’s colonies after using *T. polium* and placebo mouthwashes during the study. As a final point, a standard AB/BA crossover model was used to find the results of treatment and the carryover effect on the residual biological effects after using the mouthwash until the final phase [22]. The significant level was 0.05 in this experiment.

### Ethical considerations

Ethics Committee of the Rafsanjan University of Medical Sciences approved the research’s ethics. Approval number is 937/9/31 with IRCT code number of IRCT2013121815842N1. Participants were asked to sign a written informed consent.

### Consolidated standards of reporting trials (CONSORT) flow diagram

**Fig. 2:**
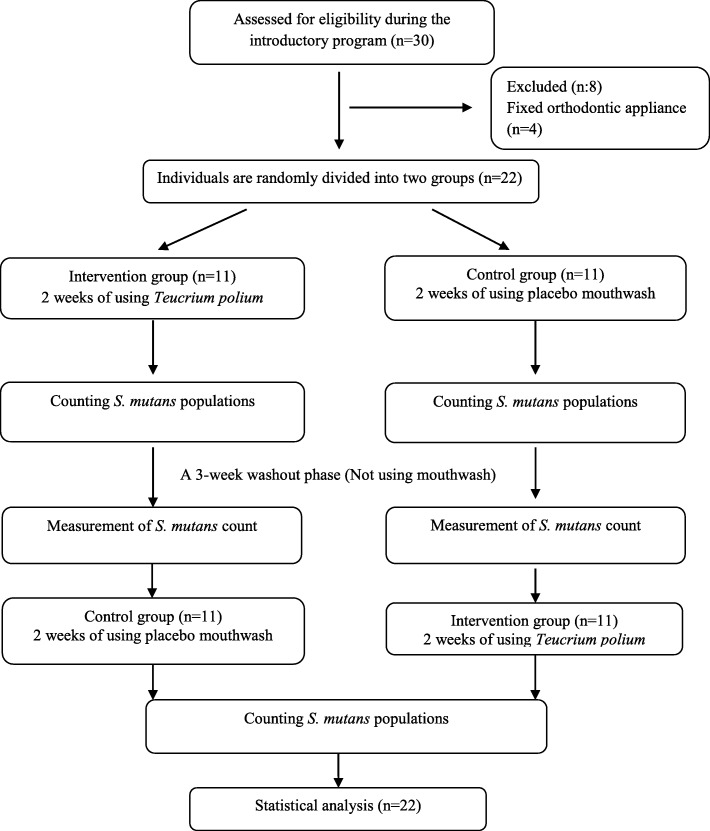
CONSORT flow diagram

CONSORT flow diagram is seen in Fig. 2.

## Results

Twenty-two dental students (11 male and 11 female students) participated in this research. The average age of participants was 23 (21 to 26 years old). The age difference between the two groups was not significant. The first group was the participants who used *T. polium* mouthwash in the first phase, and then used the placebo mouthwash during the second phase. On the other hand, the second group used the *T. polium* and placebo mouthwash conversely. The first phase lasted for two weeks, and it was followed by a three-week washout period. The second phase commenced after the washout period and lasted for two weeks. The results showed that there was no statistical difference between the numbers of *Streptococcus mutans*’s colonies per one milliliter of saliva in the groups before using the mouthwashes (Table 1).

**Table 1:** Variables in both A and B groups before starting the study

Variables			groups	
			A	B
Gender	Male	Count (percent)	5 (45.5%)	6 (54.5%)
	Female	Count (percent)	6 (54.5%)	5 (45.5%)
Age	Mean year		23.09(±1.64)	23.18(±1.78)
_Salivas’*S. mutans*_population before starting the trial	Mean(*10^6^ CFU/mL)		7.24 (±1.69)	7.86(±1.28)

**Fig. 3:**
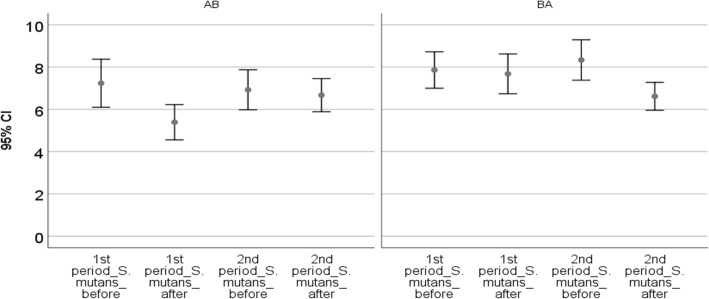
Colony count of *Streptococcus mutans* before and after each phase

In the first phase, *T. polium* mouthwash significantly pulled down the number of *S. mutans*’ colonies (*P* < 0.001). Nonetheless, placebo mouthwash did not significantly decrease the number of *S. mutans*’ colonies (*P* = 0.340). Moreover, the change in mean (decrease) in *S. mutans’* colonies was significantly higher in *T. polium* mouthwash than placebo mouthwash (P < 0.001) (Fig. 3, Table 2).

**Table 2:** Colony count of *Streptococcus mutans* before and after of each phase, and significances of effectiveness of using mouthwashes intergroups and intragroups

		Group 1	Group 2	*p*-value *
Treatment in the first phase		Treatment A	Treatment B	
1st period_S.mutans_before	Mean	7.24(±1.69)	7.86(±1.28)	
1st period_S.mutans_after	Mean	5.39(±1.24)	7.68(±1.40)	
Difference of S.mutans before and after the use of mouthwash in first phase	Mean	1.85(±0.87)	0.18(±0.60)	< 0.001
p-value in treatment		< 0.001	=0.340	
Treatment in the second phase		Treatment B	Treatment A	
2nd period_S.mutans_before	Mean	6.93(±1.41)	8.34(±1.42)	
2nd period_S.mutans_after	Mean	6.67(±1.17)	6.62(±0.98)	
Difference of S.mutans before and after the use of mouthwash in second phase	Mean	0.25(±0.74)	1.72(±1.09)	=0.001
*p*-value in treatment		=0.279	< 0.001	

* *p*-value in decreasing the colonies between two groups at the end of each period

At the second phase, after three weeks of washout period (without using the mouthwashes), the first group used the placebo mouthwash, while the second group used the *T. polium* mouthwash. Placebo mouthwash, again, did not significantly decrease the number of *S. mutans’* colonies (*P* = 0.279). The use of *T. polium* mouthwash resulted in a significant reduction in the number of *S. mutans’* colonies (*P* < 0.001). Additionally, the mean change (decrease) in the number of *S. mutans’* colonies in the *T. polium* group was significantly higher than the placebo group (*P* = 0.001) (Table 2).

Finally, the standard AB/BA crossover model analysis was conducted to find the carryover effect. The difference between the change in *S. mutans* colonies before and after using mouthwashes in the first phase (Y1) and the second phase (Y2) were calculated. This difference was significant (*P* < 0.001). It showed that an effective treatment or carryover effect existed between the treatments. To find out where the effect came from (due to the treatment or carryover effect), the second part of this analysis was conducted. In this part the sum of Y1 and Y2 was analyzed, and showed no significant differences, which approves that there is no carryover effect. Comparison of *T. polium* and placebo mouthwashes using crossover analysis showed that *T. polium* mouthwash significantly reduced the number of *S. mutans*’ colonies (P < 0.001) (Additional file 2).

## Discussion

Tooth decay is an epidemic problem in the world. While the chemical mouthwashes have their own side effects, including discolorations of tongue and tooth surfaces, alteration in taste perception, soft tissue irritations and burning sensation [23], herbal mouthwashes seem to be an appropriate substitute [24–26]. Results of this study proved that a mouthwash containing *T. polium* can effectively decrease *S. mutans* population in the mouth, and this effect can last for at least three weeks after using the mouthwash.

During the first phase of the current research, the participants noticed a tangible difference in the taste of the mouthwash containing *T. polium,* due to their treatment. It is worthy to mention this limitation was predicted before the study was conducted and several steps were designed to reduce its impacton the outcomes. Firstly, the participants voluntarily contributed to the study and the importance of following the study protocol was discussed with them. In addition, since the participants were selected from dental students (not from a general demographics), they were more likely to cooperate with the research team and understood the drawbacks. Secondly, the study was designed as an AB/BA crossover study, and a standard AB/BA crossover statistical model was utilized to analyze the outcomes and test the possible carryover effects.

*S. mutans* is considered to be the most important and pathogenic decay microorganism and it plays a major role in the onset of caries [27]. In the present study, the effect of *T. polium* mouthwash on saliva’s *S. mutans* was investigated. The design of the study in crossover model has been considered as its strong point, since its two-step design (two phases) increased its accuracy. The positive results of the two phases emphasize the efficacy of the antibacterial properties of the polei-gamander plant on *S. mutans*. In this cross-over study, two positive effects of *T. polium* mouthwash were observed: A reduction in the number of *S. mutans* colonies during the time when *T. polium* mouthwash was used and the continuous antimicrobial effect of the mouthwash in the washout period. In the Intervention group, there was a significant reduction in *S. mutans* in both phases, while in the control group, only a slight decrease inthe microbial colonieswas obtained due to the regular use of mouthwash. The counting of microbial colonies of saliva before and after each phase depicts the capability of the contributions this research has made.

In studies that investigate the antimicrobial effects of mouthwashes, the duration of the washout period is usually two to three weeks [26, 28]. In the current study, the washout period was three weeks after which the *S. mutans* value did not return to the initial level. This indicates a potent antibacterial effect of the *T. polium* mouthwash. Consequently, at the end of the second phase, when both groups had used *T. polium* mouthwash, similar results of a reduction in the microbial colony were observed. Using standard AB/BA crossover model analysis [22] indicated that there were no carryover effects.

Studies on antibacterial activity of herbal plants have shown different results. The findings of this research demonstrate that *T. polium* mouthwash, compared to the placebo mouthwash, can significantly reduce the rate of salivary *S. mutans*. Mehta et al (2013) showed that a commercial herbal mouthwash had similar effect on reducing the salivary *S. mutans* as chlorhexidine (*P* = 0.639) [26]. Shah et al.(2018) had a randomized controlled pilot study on comparing an herbal mouthwash and a mouthwash containing 0.2% chlorhexidine on reducing salivary *S. mutans*. Their herbal mouthwash containing twelve herbal plants was significantly more effective than the 0.2% chlorhexidine mouthwash in reducing oral *S. mutans* population (*P* < 0.001). They also illustrated that their herbal mouthwash had no side effects on oral or dental tissues [25]. Furthermore, a systematic review in 2018 indicated that herbal products exerted almost comparable antibacterial effect against *S. mutans* comparing with chlorhexidine [29]. Haffajee et al. [30] found that herbal mouthwashescan be effective in preventing the growth of oral microbes and can be useful in controlling dental plaques and inflammation of the gum. The range of effectiveness of antibacterial effect for polei-gamander plant has been widely reported in different studies. Mosadegh et al. [31] reported a weak antibacterial effect of ethanolic extract of polei-gamander plant on *Staphylococcus*, *Micrococcus luteus*, and *Escherichia coli,* while the antibacterial effect in our research was strong. The differences in such reports might be due to various types of bacteria, and different extraction methods.

Phenolic compounds which are present in *T. polium* herb could be the factor which is reducing the number of salivary *S. mutans*. Bravo showed that gram-positive and gram-negative bacteria are sensitive to the phenolic compounds, which are widely distributed substances in many plants [32]. Antimicrobial effects of these compounds depend on the number and the position of the phenol hydroxyl groups. It has been claimed that the toxicity effect of these compounds on the micro-organisms is directly correlated with the numbers of hydroxyl groups [33]. Another antimicrobial component existing in *T. polium* herb is tannin compound. Antimicrobial effects of these compounds are due to both suppression of tenacity of the microbes and blockage of microbial enzymatic activity [34].

Our study exhibited a positive result, nonetheless, the effects of *T. polium* mouthwash has not been compared to chlorhexidine as a gold standard. It is highly suggested to investigate and to compare the effect of polei-gamander plant’s active ingredients on different bacterial strains with that of chlorhexidinein the upcoming researches.

## Conclusion

The results of the current research indicate the aqueous extract of *T. polium* significantly reduces the colonization of *S. mutans* in human saliva. This decline was visible even after three weeks of washout period. Overall, it can be concluded the use of *T. polium* mouthwash, on a periodic basis, can reduce the risk of tooth decays. These findings can also contribute to the ingredients of other oral hygiene materials like toothpastes or chewing gums.

## Supplementary information

**Additional file 1.**

**Additional file 2.** Standard AB/BA crossover model analysis.

## Data Availability

Data of this study is attached as a Microsoft excel file.

## Abbreviations

*S. mutans*: *Streptococcus mutans*
*T. polium*: *Teucrium polium*
IRCT: Iranian Registry of Clinical Trials
TYCSB: Tryptone-Yeast-Cysteine-Sucrose-Bacitracin
VP: vogues-proskauer
CFU: colony forming unit
CONSORT: Consolidated Standards of Reporting Trials
