# Supplementary material for: The effect of antimicrobial activity of Teucrium Polium on Oral Streptococcus Mutans: a randomized cross-over clinical trial study
Source: BMC Oral Health. 2020 May 1;20:130. doi: 10.1186/s12903-020-01116-4 (PMC7195746; doi:10.1186/s12903-020-01116-4)
Supplement: Supplementary file 2 — Additional file 2. Standard AB/BA crossover model analysis. [file 12903_2020_1116_MOESM2_ESM.docx]

Table 3. Standard AB/BA crossover model analysis

| **groups** |  | **N** | **Mean(*10^6^ CFU/mL)** | **Std. Deviation** | **Std. Error Mean** |
| --- | --- | --- | --- | --- | --- |
| y1- y2 | AB | 11 | -1.5909 | 1.25495 | 0.37838 |
|  | BA | 11 | 1.5364 | 1.22904 | 0.37057 |

|  |  | F^*^ | Sig. | t-test | df | Sig. (2-tailed) | Mean Difference | Std. Error Difference | 95% Confidence Interval of the Difference | |
| --- | --- | --- | --- | --- | --- | --- | --- | --- | --- | --- |
|  |  |  |  |  |  |  |  |  | Lower | Upper |
| y1- y2 | Equal variances assumed | 0.146 | 0.707 | -5.905 | 20 | 0.000 | -3.12727 | 0.52962 | -4.23204 | -2.02251 |
|  | Equal variances not assumed |  |  | -5.905 | 19.991 | 0.000 | -3.12727 | 0.52962 | -4.23207 | -2.02248 |

* Levene's Test for Equality of Variances

| groups |  | N | Mean | Std. Deviation | Std. Error Mean |
| --- | --- | --- | --- | --- | --- |
| y1+ y2 | AB | 11 | 2.1000 | 1.01587 | 0.30630 |
|  | BA | 11 | 1.9000 | 1.25379 | 0.37803 |

|  |  | F | Sig. | t | df | Sig. (2-tailed) | Mean Difference | Std. Error Difference | 95% Confidence Interval of the Difference | |
| --- | --- | --- | --- | --- | --- | --- | --- | --- | --- | --- |
|  |  |  |  |  |  |  |  |  | Lower | Upper |
| y1+ y2 | Equal variances assumed | 0.433 | 0.518 | 0.411 | 20 | 0.685 | 0.20000 | 0.48655 | -0.81492 | 1.21492 |
|  | Equal variances not assumed |  |  | 0.411 | 19.175 | 0.686 | 0.20000 | 0.48655 | -0.81772 | 1.21772 |

* Y1: Difference of *S. mutans* before and after the use of mouthwash in first phase

** Y2: Difference of *S. mutans* before and after the use of mouthwash in second phas
